# Supplementary material for: Prolonged Lifespan, Improved Perception, and Enhanced Host Defense of Caenorhabditis elegans by Lactococcus cremoris subsp. cremoris
Source: Microbiol Spectr. 2022 May 16;10(3):e00454-21. doi: 10.1128/spectrum.00454-21 (PMC9241934; doi:10.1128/spectrum.00454-21)
Supplement: SUPPLEMENTAL FILE 1 — Supplemental material. Download spectrum.00454-21-s001.pdf, PDF file, 0.1 MB [file spectrum.00454-21-s001.pdf]

Supplementary Table 1. Mean and maximum survival days of worms fed FC or EPS

Worm Strain	Food	No. of trial (Fig. No.)	Mean survival time ± SE (days old)	Maximum survival time ± SE (days old)	No. of nematodes(no. of nematodes lost)	Logrank test *p* value
N2	OP50	1 (Fig. 1A)	11.30 ± 0.49	17.00 ± 0.29	79 (3)	6.82E-06
	FC		14.74 ± 0.39	18.42 ± 0.38	85 (3)	
N2	OP50	2	10.60 ± 0.58	15.83 ± 0.26	78 (0)	3.21E-12
	FC		15.21 ± 0.51	19.17 ± 0.28	79 (1)	
N2	OP50	3	12.22 ± 0.46	18.00 ± 0.42	79 (11)	6.78E-17
	FC		19.77 ± 0.65	23.67 ± 0.72	61 (29)	
N2 vs. *Δdaf-16*	N2-OP50	1	13.74 ± 0.43	19.12 ± 0.38	87 (0)	2.67E-05
	N2-FC		15.31 ± 0.50	20.50 ± 0.54	94 (5)	
	Δdaf-16 -OP50		9.78 ± 0.25	13.14 ± 0.31	95 (0)	2.92E-17
	Δdaf-16 -FC		15.42 ± 0.51	20.58 ± 0.08	85 (29)	
N2 vs. *Δdaf-16*	N2-OP50	2 (Fig. 5A)	13.53 ± 0.48	19.93 ± 0.23	95 (0)	1.75E-07
	N2-FC		17.67 ± 0.40	22.79 ± 0.37	90 (12)	
	Δdaf-16 -OP50		10.83 ± 0.44	16.21 ± 0.29	90 (0)	1.88E-07
	Δdaf-16 -FC		14.68 ± 0.61	19.63 ± 0.30	55 (25)	
Δdaf-16	OP50	3	8.50 ± 0.36	12.50 ± 0.25	79 (4)	9.51E-11
	FC		12.45 ± 0.52	17.00 ± 0.22	66 (11)	
Δdaf-16	OP50	4	9.50 ± 0.32	13.42 ± 0.31	88 (3)	2.76E-12
	FC		14.86 ± 0.81	20.38 ± 0.35	56 (28)	
N2 vs. *Δskn-1*	N2-OP50	1	12.72 ± 0.53	20.30 ± 0.64	98 (2)	6.60E-12
	N2-FC		18.45 ± 0.47	23.33 ± 0.61	100 (12)	
	Δskn-1- OP50		10.62 ± 0.54	17.35 ± 0.55	85 (4)	0.810
	Δskn-1 -FC		10.51 ± 0.59	18.19 ± 0.64	89 (4)	
N2 vs. *Δskn-1*	N2-OP50	2 (Fig. 5A)	13.53 ± 0.48	19.93 ± 0.23	95 (0)	1.75E-07
	N2-FC		17.67 ± 0.40	22.79 ± 0.37	90 (12)	
	Δskn-1- OP50		11.31 ± 0.52	18.29 ± 0.45	90 (0)	0.458
	Δskn-1 -FC		11.90 ± 0.52	19.50 ± 0.50	90 (5)	
Δskn-1	OP50	3	13.55 ± 0.57	21.08 ± 0.36	83 (4)	0.548
	FC		13.11 ± 0.62	20.04 ± 0.65	70 (12)	
Δskn-1	OP50	4	14.71 ± 0.62	21.06 ± 0.34	61 (4)	0.430
	FC		15.14 ± 0.67	21.61 ± 0.45	58 (10)	
Δskn-1	OP50	5	9.65 ± 0.49	16.04 ± 0.24	86 (4)	0.631
	FC		8.17 ± 0.72	15.94 ± 0.47	61 (19)	

N2 vs. *Δpmk-1*	N2-OP50	1	13.74 ± 0.43	19.12 ± 0.38	87 (0)	2.67E-05
	N2-FC		15.31 ± 0.50	20.50 ± 0.54	94 (5)	
	Δpmk-1- OP50		9.75 ± 0.35	14.63 ± 0.46	103 (0)	3.39E-05
	Δpmk-1 -FC		11.07 ± 0.64	18.58 ± 0.43	83 (26)	
N2 vs. *Δpmk-1*	N2-OP50	2 (Fig. 5A)	13.53 ± 0.48	19.93 ± 0.23	95 (0)	1.75E-07
	N2-FC		17.67 ± 0.40	22.79 ± 0.37	90 (12)	
	Δpmk-1- OP50		10.39 ± 0.43	15.93 ± 0.47	93 (0)	8.67E-07
	Δpmk-1 -FC		13.35 ± 0.62	20.73 ± 0.30	85 (14)	
Δpmk-1	OP50	3	10.44 ± 0.53	16.58 ± 0.23	80 (0)	2.91E-04
	FC		11.97 ± 0.69	19.96 ± 0.33	89 (2)	
Δpmk-1	OP50	4	11.57 ± 0.37	15.58 ± 0.35	84 (0)	0.006
	FC		12.90 ± 0.40	17.33 ± 0.30	82 (8)	
Δpmk-1	OP50	5	10.90 ± 0.55	16.92 ± 0.23	80 (0)	0.002
	FC		11.93 ± 0.86	20.30 ± 0.44	67 (13)	
Δpmk-1	OP50	6	9.22 ± 0.52	15.35 ± 0.36	86 (4)	1.98E-06
	FC		11.85 ± 0.95	20.63 ± 0.64	54 (38)	
N2	OP50	1	12.66 ± 0.47	18.27 ± 0.54	87 (3)	1.90-E12 (OP50 vs. FC)
	FC		17.66 ± 0.54	22.83± 0.35	86 (4)	
	Heat killed OP50		14.99 ± 0.49	21.12 ± 0.43	92 (8)	1.86-E06 (Heat killed OP50 vs. Heat killed FC)
	Heat killed FC		18.83 ± 0.64	29.42 ± 1.28	81 (19)	
N2	OP50	2 (Fig. 1B)	12.16 ± 0.49	17.93 ± 0.29	94 (6)	1.60-E14 (OP50 vs. FC)
	FC		17.78 ± 0.39	21.25± 0.56	80 (20)	
	Heat killed OP50		15.03 ± 0.43	20.57 ± 0.30	96 (12)	0.002 (Heat killed OP50 vs. Heat killed FC)
	Heat killed FC		17.50 ± 0.48	24.50 ± 0.88	91 (18)	
N2	Heat killed OP50	3	14.01 ± 0.49	19.65 ± 0.37	88 (2)	2.71E-05
	Heat killed FC		16.81 ± 0.67	24.05 ± 0.39	77 (23)	
N2	H_2O liposome	1	10.08 ± 0.55	17.25 ± 0.37	79 (3)	0.422
	EPS liposome		11.56 ± 0.43	16.67 ± 0.37	78 (2)	
N2	H_2O liposome	2	9.34 ± 0.52	15.65 ± 0.32	87 (2)	0.054
	EPS liposome		11.19 ± 0.47	16.88 ± 0.37	88 (1)	
N2	H_2O liposome	3 (Fig. 1H)	9.98 ± 0.47	15.00 ± 0.47	77 (5)	0.009
	EPS liposome		11.54 ± 0.57	18.05 ± 0.79	74 (6)	

Supplementary Table 2. Mean and maximum survival days of worms infected with *Salmonella enterica* serovar Enteritidis or *Staphylococcus aureus*

Worm Strain	Food	Pathogens	No. of trial (Fig. No.)	Mean survival time ± SE (days)	Maximum survival time ± SE (days)	No. of nematodes (no. of nematodes lost)	Logrank test *p* value
N2	OP50<br>FC	*S. enterica*	1<br>(Fig. 3A)	5.67 ± 0.28<br>8.05 ± 0.30	10.29 ± 0.42<br>12.50 ± 0.44	90 (0)<br>89 (1)	1.27E-05
N2	OP50<br>FC	*S. enterica*	2	5.61 ± 0.29<br>6.91 ± 0.28	10.23 ± 0.27<br>10.86 ± 0.34	70 (0)<br>70 (0)	0.021
N2	OP50<br>FC	*S. enterica*	3	5.41 ± 0.27<br>6.86 ± 0.23	9.42 ± 0.34<br>9.83 ± 0.22	79 (1)<br>80 (0)	5.17E-03
N2	OP50<br>FC	*S. aureus*	1<br>(Fig. 3A)	4.05 ± 0.22<br>6.23 ± 0.37	7.27 ± 0.51<br>11.75 ± 0.95	84 (1)<br>78 (2)	9.65E-06
N2	OP50<br>FC	*S. aureus*	2	5.17 ± 0.29<br>7.33 ± 0.48	9.96 ± 0.48<br>14.42 ± 0.52	88 (2)<br>84 (6)	1.15E-04
N2	OP50<br>FC	*S. aureus*	3	3.88 ± 0.27<br>5.31 ± 0.33	8.50 ± 0.57<br>10.73 ± 0.74	88 (4)<br>88 (5)	4.64E-03
N2 vs.<br>*Δskn-1*	N2-OP50<br>N2-FC<br>Δ*skn-1* -OP50<br>*Δskn-1* -FC	*S. enterica*	1<br>(Fig. 5D)	5.63 ± 0.21<br>7.98 ± 0.30<br>5.24 ± 0.26<br>5.04 ± 0.26	8.21 ± 0.27<br>12.14 ± 0.29<br>9.79 ± 0.16<br>8.81 ± 0.31	90 (0)<br>90 (0)<br>90 (0)<br>90 (0)	2.32E-08<br><br>0.688
N2 vs.<br>*Δskn-1*	N2-OP50<br>N2-FC<br>*Δskn-1* -OP50<br>*Δskn-1* -FC	*S. enterica*	2	6.11 ± 0.25<br>8.63 ± 0.25<br>4.94 ± 0.29<br>6.05± 0.29	9.67 ± 0.11<br>11.67 ± 0.49<br>9.33 ± 0.32<br>10.42 ± 0.40	80 (5)<br>80 (0)<br>80 (0)<br>80 (0)	1.53E-07<br><br>0.042
Δskn-1	OP50<br>FC	*S. enterica*	3	7.51 ± 0.37<br>6.55 ± 0.34	13.36 ± 0.33<br>11.96 ± 0.54	92 (0)<br>87 (0)	0.091
N2 vs.<br>*Δskn-1*	N2-OP50<br>N2-FC<br>*Δskn-1* -OP50<br>*Δskn-1* -FC	*S. aureus*	1	4.97 ± 0.24<br>8.29 ± 0.41<br>4.91 ± 0.29<br>5.73 ± 0.33	8.35 ± 0.30<br>15.19 ± 0.29<br>10.00± 0.27<br>11.19 ± 0.47	88 (2)<br>86 (4)<br>90 (0)<br>86 (1)	1.17E-08<br><br>0.114
N2 vs.<br>*Δskn-1*	N2-OP50<br>N2-FC<br>*Δskn-1* -OP50<br>*Δskn-1* -FC	*S. aureus*	2<br>(Fig. 5D)	6.25 ± 0.30<br>8.44 ± 0.43<br>5.16 ± 0.29<br>4.93 ± 0.28	10.81 ± 0.52<br>15.00 ± 0.65<br>9.92 ± 0.57<br>9.33 ± 0.55	89 (5)<br>80 (12)<br>80 (0)<br>81 (1)	2.00E-04<br><br>0.690
Δskn-1	OP50<br>FC	*S. aureus*	3	6.13 ± 0.33<br>7.10 ± 0.36	11.27 ± 0.38<br>12.65 ± 0.74	87 (0)<br>85 (0)	0.143
